# Descriptive study of 896 Oral squamous cell carcinomas from the only University based Oral Pathology Diagnostic Service in Sri Lanka

**DOI:** 10.1186/s12903-015-0139-y

**Published:** 2016-01-08

**Authors:** Primali Rukmal Jayasooriya, Thushara Nilmini Pitakotuwage, Balapuwaduge Ranjit Rigorbert Nihal Mendis, Tommaso Lombardi

**Affiliations:** Department of Oral Pathology, Faculty of Dental Sciences, University of Peradeniya, Peradeniya, Sri Lanka; Laboratory of Oral and Maxillofacial Pathology, Unit of Oral Medicine & Division of Oral and Maxillofacial Surgery, Department of Surgery, University Hospitals of Geneva & University of Geneva, 19, rue Barthelemy-Menn, 1205 Geneva, Switzerland

**Keywords:** Oral squamous cell carcinoma, Metastasis, Neck dissection

## Abstract

**Background:**

The main objective of this study was to describe selected clinico-pathological characteristics of Oral Squamous Cell Carcinoma (OSCC) in Sri-Lanka.

**Materials & methods:**

The study sample comprised of eight hundred and ninety six biopsies diagnosed as OSCC. The clinical and histopathological features were analyzed using the Chi-square test.

**Results:**

Of the 896 biopsies, 801 were primary OSCCs, while 95 were recurrent OSCCs. Majority of the patients (78 %) were in the 5^th^ to 7^th^ decades of life and showed a male predilection. The buccal mucosa was the commonest site of primary OSCC comprising of 43 % of the sample. Of the primary OSCCs, with known TNM stage, 86 % were in stage 3&4 and majority (59 %) of stage 4 tumours showed tumour at one or more excision margins. Of the recurrent OSCC, 46 % developed their recurrences within one year of the excision of the primary tumour.

**Conclusion:**

In Sri-Lanka, OSCC is a major problem. Only half the patients had completely excised tumours (with clearance of >5 mm at all excision margins) at operation, and recurrences appeared early. This data should be considered in the future management policy of OSCC in Sri-Lanka.

## Background

According to the Sri Lankan cancer incidence data for year 2005, compiled by the National Cancer Control Programme, cancer of the lip, oral cavity and pharynx was the commonest cancer occurring in males with a crude rate of 12.7 per 1,00,000 population. On the other hand, in females it is less common than cancer of the breast, cervix, ovary, thyroid and esophagus with a crude rate of 3.8 per 100,000 populations [1]. In addition, Sri Lanka also has one of the highest oral cancer mortality rates in the world.

Majority of the cancers that occur in the oral cavity are oral squamous cell carcinomas (OSCC) arising from the stratified squamous epithelial lining of the buccal mucosa, tongue, floor of the mouth, palate and lip. Tobacco use, either in the form of cigarettes, cigars, pipes and beedi or smokeless tobacco used for betel chewing has been without doubt shown to be the main culprit of OSCC [2, 3]. Even with this knowledge, in Sri Lanka, OSCC is one of the major health problems as both males and females from rural areas of the country traditionally chew betel quid, which contains areca nut, tobacco, lime paste and spices wrapped in betel leaf [4]. In addition, betel chewing males also smoke and take alcohol, which further increases the susceptibility to develop OSCC.

The clinical outcome of patients with OSCC is assessed based on tumour-node-metastasis (TNM) system and at present it is the most reliable indicator on which therapeutic decisions are made. According to TNM staging, stage I or stage II disease defines a relatively small primary tumour with no nodal involvement. Stage III and stage IV includes large primary tumours, which may invade into underlying structures and/or spread to regional lymph nodes [5–7].

The main objective of this study was to describe selected clinico-pathological data for primary and recurrent OSCCs reported at the only University based Oral Pathology Diagnostic Service in Sri Lanka during a 2 years and nine month period, in order to highlight the magnitude of problem.

## Method

Archives of the Department of Oral Pathology revealed a total of eight hundred and ninety six biopsies diagnosed as Oral squamous cell carcinomas during a two years and nine month period from 2009 to 2011 September. Out of the 896 biopsies, 801 were primary OSCCs, while 95 were recurrent OSCCs.

Data such as the patient’s age, sex, site of the lesion and TNM staging were obtained from the biopsy request forms, while the histopathological diagnosis, status of the excision margins and lymph node involvement were obtained from the biopsy reports. Statistical analysis was performed using the Chi-square test.

Ethical clearance to analyze clinico-pathological information of the oral squamous cell carcinoma patients was obtained from the Dental Faculty, Ethics and review committee of the University of Peradeniya, Sri Lanka. The information was obtained retrospectively from biopsy request forms and oral pathology data base maintained in the department and the identity of each patient is not disclosed in the study. Therefore, according to the existing guidelines, it is not necessary to obtain written or verbal consent from each patient.

## Results

The study sample comprised of 77 % of Sinhalese, 19 % of Tamil and 4 % other ethnic groups. Approximately 68 % of the patients lived in rural areas while only 32 % were from urban and semi urban areas of the country. Table 1 shows the selected clinico-pathological characteristics of patients presenting with primary OSCC. Accordingly, the youngest patient presenting with OSCC was 20 years of age while the oldest patient was 93 years of age. In addition majority of the patients (78 %) were in the 5^th^ to 7^th^ decades of life. Approximately 71.5 % of the study sample comprised of males with a female to male ratio of 1:2.7. The buccal mucosa was the commonest site of primary OSCC comprising of 43 % of the sample, followed by 23 % with OSCC of the tongue. Twenty seven percent of the study sample had large primary tumours, which involved two or multiple sites. With reference to clinical presentation, most primary OSCCs presented as ulcers or exophytic growths. Most primary OSCCs were sub classified as early invasive depending on the depth of tumour invasion and well, moderate or poorly differentiated OSCC depending on the degree of differentiation.

**Table 1:** Selected clinico-pathological characteristics of patients presenting with primary OSCC

Feature	Number of patients (%) *n* = 801
Age	
<30–40	28 (03.5)
41–50	132 (16.5)
51–60	257 (32.1)
61–70	219 (27.3)
>70	155 (19.3)
unknown	10 (01.3)
Sex	
Male	573 (71.5)
Female	212 (26.5)
unknown	16 (02.0)
Site	
Buccal mucosa	349 (43.6)
Tongue	190 (23.7)
Floor of the mouth	66 (08.2)
Alveolar mucosa	103 (12.9)
Palate	73 (09.1)
Lip	20 (02.5)
Number of sites involved	
Single	581 (72.5)
Two	71 (08.9)
Three or more than three	149 (18.6)
Clinical presentation	
Ulcer	408 (50.9)
White lesion	57 (07.1)
Red/white mixed lesion	33 (04.1)
Exophytic growth	303 (37.9)
Type of Biopsy	
Incision	488 (60.9)
Excision	313 (39.1)
Pathology	
Early invasive SCC	111 (13.9)
WDSCC	360 (44.9)
MDSCC	218 (27.2)
PDSCC	37 (04.6)
Verrucous Carcinoma	15 (01.9)
Basaloid squamous cell carcinoma	27 (03.4)
Other	33 (04.1)

Table 2 also shows the type of surgical intervention, histopathological characteristics of excision margins and lymph node involvement of patients with primary OSCC who have undergone definitive surgical treatment with curative intent, when graded according to TNM stage. Although, majority of the patients (92 %) had received neck dissections, in addition to local excision, only 25 % presented with lymph node metastasis. In addition, only 51 % of the patients had completely excised primary tumours.

**Table 2:** Characteristics of the treatment received by the patients, and pathological findings

Characteristics of treatment received by patients	TNM Stage 1	TNM Stage 2	TNM Stage 3	TNM Stage 4	Unknown
Total number of patients at each TNM stage (%)	20 (6.3)	19 (6.1)	117 (37.4)	122 (39.0)	35 (11.2)
Type of surgery					
Excision of the lesion only	20	03	----	---	----
Excision of the lesion + Neck dissection	--	16	117	122	35
*Status of the surgical margins of the lesion					
Tumour with >5 mm clearance	14(70)	15(79)	71(60.6)	50(41)	12
Tumour with <1 mm clearance	--	---	24(20.5)	46(37.8)	09
Tumour with 1–5 mm clearance	02(10)	---	14(12.0)	13(10.6)	---
Tumour with dysplasia at excision margin/s	04(20)	04(21)	08(06.9)	13(10.6)	14
Status of the lymph nodes					
Metastasis present	---	01(6.3)	31(26.5)	39 (32)	04
Metastasis absent	----	15(93.7)	86(73.5)	83 (68)	31

Tumour with >5 mm clearance at all excision margins are considered as completely excised

Tumour with <1 mm clearance at one or more than one excision margin are considered as incompletely excised

Tumour with 1–5 mm clearance at one or more than one excision margin are considered as closely excised

Tumour with dysplasia at excision margins considered as such when any degree of dysplasia extends in to one or more than one excision margin

Table 3 shows some selected clinico-pathological characteristics of recurrent OSCC. Except for a single patient all the others who presented with recurrences were over 40 years of age. In addition, there were no statistically significant differences in the gender distribution, between the patients presenting with primary and recurrent OSCCs (*p* > 0.05). Majority (46 %) developed their recurrences within one year of the excision of the primary tumour while 27 % had three years or more disease free period before the recurrence.

**Table 3:** Selected clinico-pathological characteristics of patients presenting with recurrent OSCC

Feature	Number of patients (%) *n* = 95
Age	
<40 years	01 (01.1)
>41 years	94 (98.9)
Sex	
Male	69 (72.6)
Female	26 (27.4)
Site of Primary cancer	
Buccal mucosa	42 (44.2)
Tongue	15 (15.8)
Other	38 (40.0)
Tumour free period after treatment	
<1 year	44 (46.3)
1–2 years	24 (25.3)
3–4 years	11 (11.6)
>5 years	16 (16.8)

Table 4 shows a comparison of sex and site distribution of patients who are younger and older than 40 years. Accordingly, patients who were younger than 40 years tend to present with OSCC of the tongue (*P* = 0.00063) or floor of the mouth (*P* = 0.00031) more often compared to patients who were older than 40 years. However, no statistically significant changes were observed between the ratios of gender distribution in the two groups (*p* < 0.05).

**Table 4:** Comparison of sex and site distribution of patients presenting with primary OSCC who are younger and older than 40 years

	<40 years (%)	>40 years (%)	Chi square test
Sex			
Male	21 (75.0)	544 (72.82)	NS (*P* = 0.79)
Female	07 (25.0)	203 (27.18)	
Total	28	747	
Site			
Buccal mucosa	03 (10.7)	341 (44.70)	Buccal mucosa *vs* tongue
Tongue	11 (39.2)	178 (23.33)	*P* = 0.00063
Floor of the mouth	05 (17.8)	61 (07.99)	Buccal mucosa vs floor of the mouth
Other	09 (32.3)	183 (23.98)	*P* = 0.00031
Total	28	763	

When the relationship between the site of occurrence of the primary OSCC and presence or absence of lymph node metastasis was analyzed, (Table 5), primary tumours which occurred on the tongue (*P* = 0.0029) and the floor of the mouth (*P* = 0.0026) showed a statistically significant higher tendency of developing lymph node metastasis compared to primary tumours of buccal mucosa. However, no significant differences were observed when lymph node metastasis rates were compared between tongue and floor of the mouth.

**Table 5:** The relationship between the site and presence/absence of lymph node metastasis

Primary site	Lymph node metastasis present (*n* = 75)(%)	Lymph node metastasis absent (*n* = 215)(%)	Chi square test
Buccal mucosa (BM)	21 (16.0)	109 (84.0)	BM vs T (*p* = 0.0029)
Tongue (T)	20 (37.7)	33 (62.3)	BM vs FOM (*p* = 0.0026)
Floor of the mouth (FOM)	12 (46.2)	14 (53.8)	BM vs AM, P, L (NS)
Alveolar mucosa (AM)	11 (29.7)	26 (70.2)	FOM vs T (NS)
Palate (P)	07 (24.1)	22 (75.9)	
Lip (L)	04 (26.6)	11 (73.4)	

Primary tumours which extend to multiple sites do not seem to provide additional difficulty in obtaining complete clearance compared to tumours involving a single site (Table 6). However, neck nodes containing metastatic tumours is observed more often in patients who have large primary tumours which extend in to multiple sites compared to tumours which involve a single site only (*P* = 0.01) (Table 6).

**Table 6:** The relationship between number of sites involved by the primary tumour and status of the excision margins and lymph node metastasis

	Single site	Two sites	Multiple sites	Chi square test
Status of excision margins				
Tumour with >5 mm clearance	103(63.6)	22(50.0)	36(50.0)	
Tumour with <1 mm clearance	45(27.8)	12(27.3)	22(30.5)	Single vs two sites = NS
Tumour with dysplasia at excision margin/s	26(16.0)	08(18.2)	09(12.5)	Single vs two sites = NS
Tumour with 1–5 mm clearance	22(13.4)	02(4.5)	05 (7.0)	Two vs multiple = NS
Status of lymph nodes				
Lymph node metastasis present	41(21.1)	10(30.3)	24(38.0)	
				Single vs Multiple *p* = 0.01
Lymph node metastasis absent	153(78.9)	23(69.7)	39(62.0)	Single vs Two NS
				Two vs Multiple NS

## Discussion

Epidemiological studies have shown that betel quid chewing is a major risk factor for development of both oral and pharyngeal cancers and also oral pre-cancerous lesions [2, 4]. Earlier reports in the 1980s from Sri Lanka and a recent report from Solomon Islands [8, 9] shows a very high rate of approximately 50 % betel quid chewers in both sexes. However, a recent report from Sri Lanka [4] indicates a reduction in numbers of betel chewers, most probably the result of life style changes that have occurred over the years. In addition, a remarkable decline in the habit of smoking is also observed [10]. Accordingly, an article published by Ariyawardena et al. in 2011 [11], on oral and pharyngeal SCC incidence data over a period of 20 years obtained from hospital based cancer registry reports [1] has shown a decline. However, most experts in the field believe that under-reporting could have contributed to the above finding. Therefore, even at present Sri Lanka remains the country with the highest OSCC incidence in South East Asia [1, 12].

The Department of Oral Pathology, Faculty of Dental Sciences, University of Peradeniya, Sri Lanka is the only center in the entire country, which specializes in providing an Oral Pathology based, biopsy diagnostic reporting service. However, Pathologists with Medical degrees, also report on oral biopsies. Hence, the institution from which the present data is obtained does not report on all OSCCs detected in the country. In addition, a majority of the biopsies reported, come from the central province of Sri Lanka, where the institution is located.

Approximately, two thirds of the primary OSCCs reported from the Department of Oral Pathology was incisional biopsies, while only one third has been excisions. However, this does not reflect the true picture of the number of patients, who receive surgery with a curative intent, as some of the patients may have had excisions that have been reported by Pathologists attached to respective hospitals where the surgery was performed.

According to cancer incidence data for year 2005, compiled by the National cancer control programme, in Sri Lanka [1], male to female ratio of 3:1 have been observed. The male to female ratio of 2.7:1 observed in the present study is compatible with the above finding. In addition, according to the 2005 cancer incidence data [1], OSCC was shown to be more common in adults over 45 years, which is also compatible with our results. Furthermore, no discrepancies have been observed in the site distribution, with both studies reporting buccal mucosa and tongue as the commonest and 2^nd^ commonest sites where OSCCs occur. However, only 2.5 % of the total primary SCCs has been diagnosed as lip cancers in the present study, which does not reflect the true incidence of lip cancers but only the number received by our institution. Because, although, majority of the intra oral SCCs are treated by Oral and Maxillo-facial Surgeons, cancer of the lip and parotid glands may receive surgical treatment from General Surgeons with Medical degrees, who would send their biopsies to Medical Pathologists.

Previous studies on epidemiological data of OSCCs from developed and developing countries have shown differences with respect to age, gender and site distribution [13, 14]. These differences namely are as follows. In developed countries OSCCs are diagnosed more commonly in elderly patients in 7^th^ to 8^th^ decade of life while in developing countries it is more prevalent in patients in 5^th^ to 6^th^ decades of life. Generally males are more commonly affected compared to females by OSCC in both developed (male : female ratio 2.5: 1) and developing (male : female ratio 3:1) countries [1, 13]. However, the difference between gender distributions is more in developing countries, when compared with developed countries. In developed countries the difference in the gender distribution is reducing in recent years due to more females taking up tobacco related habits including smoking [13]. With reference to site distribution, although the tongue is considered as the commonest site of occurrence of primary OSCCs in developed countries, betel chewing results in cancer of the buccal mucosa more commonly in patients from developing countries. This is mainly because constituents of betel quid which is claimed to produce a sense of well being and increased capacity to work by stimulation of parasympathetic nervous sysem is kept in the buccal pouch for prolonged periods of time in order to obtain maximum effect [14].

Majority of the primary tumours comprised of conventional OSCCs, out of which 13 % were early invasive SCCs. The advantage of having an early invasive OSCC, includes, ability to obtain complete clearance without mutilative extensive surgery, which results in having a higher chance of achieving a complete cure. In addition, more aggressive poorly differentiated OSCCs, were very few in number comprising only 4 % of the total sample, most probably reflecting the contribution of etiological factors such as betel chewing towards the development of well or moderately differentiated tumours. The verrucous carcinomas have the best prognosis [15, 16], due to absence of invasion and metastasis also comprised of only 1 % of the study sample. These tumours usually occur with the use of beedi, a specific form of tobacco smoking practiced in South Asia [8]. The very low incidence of verrucous carcinoma cannot be attributed to a reduction in the risk habit as there is no decrease in the consumption of beedi reported in recent years [10]. Therefore, the fact that verrucous carcinomas that do not receive surgical treatment tends to develop in to SCCs [15, 16] may have contributed to the very low incidence identified in the present study.

In our study sample (Table 2) majority of the patients who received surgical excision of the primary tumour also received neck dissections. Some received prohylatic neck dissections while remaining neck dissections were performed with therapeutic intention. Types of neck dissections that the patients had received included radical and modified radical neck dissections [17]. In the present sample, out of the different types mentioned above most patients had received different types of selective neck dissections. The fact that majority of patients received neck dissections and the fact that approximately 45 % of the tumours were incompletely or closely excised or had dysplasia in excision margins support the fact that most patient being late presenters with large (T3/4) primary tumours. In addition, the fact that majority of our Oral and Maxillofacial Surgeons operate without the availability of frozen sections may have also contributed to the relatively large percentage of patients who ended up with incompletely excised primary tumours.

No difference in gender or site distribution was observed between primary and recurrent OSCCs in the present study sample. The most interesting finding with reference to recurrent OSCCs was the fact that majority of the tumours occurred within two years of the first surgery. In addition, all the recurrent OSCCs developed in patients with completely excised primary tumours. As there would have been at least changes that could be detected at molecular level in these patients without which the chance of developing a recurrence is very remote, investigations to identify these molecular changes would be extremely useful to prevent recurrences in the future.

The fact that younger patients develop OSCCs on the tongue and FOM in contrast to buccal mucosa suggests that some known (smoking/alcohol) and unknown aetiological factors other than betel quid chewing may play a major role in these patients [8]. The role played by these hitherto unknown factors needs further investigations.

## Conclusion

In conclusion, this study confirms previously established demographic factors such as age, gender and site distribution for OSCCs in Sri Lankan patients. In addition, the study highlights the importance of initiating more preventive and early detection strategies, due to the fact that most primary tumours at present require extensive surgery, which carries a high mortality and morbidity rate.

## Abbreviations

OSCC: oral squamous cell carcinoma
TNM: tumour-node-metastasis staging
FOM: floor of the mouth
